# Protective effect of extract of *Cordyceps sinensis *in middle cerebral artery occlusion-induced focal cerebral ischemia in rats

**DOI:** 10.1186/1744-9081-6-61

**Published:** 2010-10-19

**Authors:** Zhenquan Liu, Pengtao Li, Dan Zhao, Huiling Tang, Jianyou Guo

**Affiliations:** 1 School of Basic Medical Sciences, Beijing University of Chinese Medicine, Beijing 100029, P.R China; 2 Key Laboratory of Mental Health, Institute of Psychology, Chinese Academy of Sciences, Beijing 100101, P.R China

## Abstract

**Background:**

Ischemic hypoxic brain injury often causes irreversible brain damage. The lack of effective and widely applicable pharmacological treatments for ischemic stroke patients may explain a growing interest in traditional medicines. From the point of view of "self-medication" or "preventive medicine," *Cordyceps sinensis *was used in the prevention of cerebral ischemia in this paper.

**Methods:**

The right middle cerebral artery occlusion model was used in the study. The effects of *Cordyceps sinensis *(Caterpillar fungus) extract on mortality rate, neurobehavior, grip strength, lactate dehydrogenase, glutathione content, Lipid Peroxidation, glutathione peroxidase activity, glutathione reductase activity, catalase activity, Na^+^K^+^ATPase activity and glutathione S transferase activity in a rat model were studied respectively.

**Results:**

*Cordyceps sinensis *extract significantly improved the outcome in rats after cerebral ischemia and reperfusion in terms of neurobehavioral function. At the same time, supplementation of *Cordyceps sinensis *extract significantly boosted the defense mechanism against cerebral ischemia by increasing antioxidants activity related to lesion pathogenesis. Restoration of the antioxidant homeostasis in the brain after reperfusion may have helped the brain recover from ischemic injury.

**Conclusions:**

These experimental results suggest that complement *Cordyceps sinensis *extract is protective after cerebral ischemia in specific way. The administration of *Cordyceps sinensis *extract significantly reduced focal cerebral ischemic/reperfusion injury. The defense mechanism against cerebral ischemia was by increasing antioxidants activity related to lesion pathogenesis.

## Background

Ischemic hypoxic brain injury often causes irreversible brain damage. The cascade of events leading to neuronal injury and death in ischemia includes the release of cytokines and free radicals, and induction of inflammation, apoptosis, and excitotoxicity [1]. Reperfusion of ischemic areas could exacerbate ischemic brain damage through the generation of reactive oxygen species. The lack of effective and widely applicable pharmacological treatments for ischemic stroke patients may explain a growing interest in traditional medicines. Recently, from the point of view of "self-medication" or "preventive medicine," several dietary supplements are used in the prevention of life-style related diseases including cerebral ischemia.

Mushrooms and primarily basidiomycetous fungi are popular and valuable foods that are low in calories and high in minerals, essential amino acids, vitamins, and fibers [2]. Some of them produce substances with potential medical effects, and are called medicinal mushrooms [3]. *Cordyceps sinensis *(Caterpillar fungus) (CS) has been used as a tonic for longevity, endurance, and vitality for thousands of years by the Chinese [4]. Many studies have shown that *Cordyceps sinensis *(CS) modulates immune responses [5-7], inhibits tumor cell proliferation [8,9], enhances hepatic function [10], regulates insulin sensitivity [11], decreases plasma cholesterol levels [12], and has hypotensive and vasorelaxant activity [13]. In particular, CS modulates steroidogenesis. Huang reported that CS induced 17β-estradiol production [14]. There is strong evidence that chronic 17β-estradiol treatment has both potent and long-lasting effects on improved pathophysiological outcome after brain ischemia in experimental animal models [15,16]. Clinical studies have demonstrated that estrogens enhance mood and cognition and delay cognitive decline [17,18]. Thus, we hypothesize that *Cordyceps sinensis *possess protective effect of against ischemia-induced brain infarction by modulating 17β-estradiol production. The present study investigated the effects of *Cordyceps sinensis *on mortality rate, neurobehavior, grip strength, lactate dehydrogenase, glutathione content, Lipid Peroxidation, glutathione peroxidase activity, glutathione reductase activity, catalase activity, Na^+^K^+^ATPase activity and glutathione S transferase activity in a rat model. These data may help in the development of effective and widely applicable pharmacological treatments for ischemic stroke patients with traditional medicines.

## Materials and methods

### Animals

Healthy male adult wistar rats (2 months old and weighing 225 ± 25 g) were used in the study. This study was performed in accordance with the Guide for the Care and Use of Laboratory Animals. Care was taken to minimize discomfort, distress, and pain to the animals.

### Chemicals

#### *Cordyceps sinensis *extract (CSE) preparation

The seed of *Cordyceps sinensis *was purchased from the Agricultural Culture Collection of China. Five to six pieces of the mycelia of Cordyceps sinensis were transferred from a slant into 500 mL Erlenmeyer flasks containing 300 mL of fermented culture medium (20% potato extract liquid +2.0% dextrose +0.1% KH_2_PO_4_+0.05% MgSO_4_). The culture was incubated at 27°C on a rotary shaker at 180 rmp for 7 days [19,20].

Preparation of the CSE was as follows: 30 g of cultured *Cordyceps sinensis *mycelium powder was extracted with 240 ml of water in a water bath of 100°C for 3 h with reflux. Eighty milliliters of the water extract was then lyophilized to yield 2.9 g of the dry powder. The rest of the water extract (160 ml) was mixed with 160 ml of absolute ethanol for extraction. This 50% alcoholic fraction was dried to yield 3.7 g of the dry powder. The combination of both aqueous and alcohol extracts was then used in the present study.

### Experimental design

The animals were separated into three groups of eight rats each. The first group served as sham (SHAM). The second group was the ischemic group (MCAO). Group I and group II were treated orally by distilled water for 30 days respectively. Group III (CSE-4), Group IV (CSE-8) and Group V (CSE-10) were treated orally by CSE (4, 8 and 10 mg/kg/day respectively) for 30 days followed by MCAO induced cerebral ischemia.

The right middle cerebral artery occlusion (MCAO) was performed using an intraluminal filament model and the method described by Longa *et al*. [21]. In brief, the rats were anesthetized with chloral hydrate (400 mg/kg, i.p.), a 4-0 nylon monofilament with a blunt end was introduced into the external carotid artery (ECA) and advanced into the middle cerebral artery via the internal carotid artery (ICA) (17-20 mm), until a slight resistance was felt. Successful occlusion was confirmed by an 87-90% reduction in cerebral blood flow (CBF), as measured by laser-Doppler flowmetry [22].

Two hours after the induction of ischemia, the filament was slowly withdrawn and the animals were returned to their cages for a period of 22 hours of reperfusion. Throughout the procedure, the body temperature was maintained at 37^° ^C, with a thermostatically controlled infrared lamp. In sham rats, the ECA was surgically prepared for the insertion of the filament, but the filament was not inserted. The final number of rats was as follows: SHAM group n = 8; MCAO group n = 6; CSE-4 group n = 6; CSE-8 group n = 7 and CSE-10 group n = 8.

### Neurobehavioral test

The sensorimotor integrity was conducted to assess the neurobehavior at 24 h after MCAO in rats [23]. Five categories of motor neurological findings were scored: 0, no observable deficit; 1, forelimb flexion; 2, forelimb flexion and decreased resistance to lateral push; 3, forelimb flexion, decreased resistance to lateral push and unilateral circling; 4, forelimb flexion, unable or difficult to ambulate. Animals that showed the features of the higher scores also showed all the features of the lower grades.

### Grip strength study

Grip strength in all the animals was measured for evaluation of neuromuscular strength, as described by Ali *et al*. [24]. The neuromuscular strength tests were carried out between 9:00 a.m. to 4:00 p.m. under standard laboratory conditions.

### Tissue preparation

After grip strength measurement, blood samples were drawn from the tail vein from all the groups and serum was separated for biochemical estimations. Thereafter, the animals were sacrificed immediately and their brains were taken out to dissect the hippocampus (HIP). Post-mitochondrial supernatant (PMS) obtained from 10% homogenate of tissue was used for the estimation of various parameters related with oxidative stress.

### Biochemical estimations

In serum, lactate dehydrogenase (LDH) was estimated using a method described by Lum *et al *[25]. The PMS and HIP were used for the assay of glutathione (GSH) content, Lipid peroxidation (LPO), glutathione peroxidase (GPx) activity, glutathione reductase (GR) activity, catalase (CAT) activity, Na^+^K^+^ATPase activity and glutathione S transferase (GST) activity [26-31].

### Statistical analysis

The data are expressed as mean ± SEM. Statistical differences between means were determined by one-way analysis of variance (ANOVA), followed by Dunnett t-test. The values of *P *< 0.05 were considered as significant.

## Results and discussion

In this study, the cerebroprotective effect of *Cordyceps sinensis *extract on ischemic neuronal damage was clearly demonstrated using focal ischemia model rats.

The behavioral tasks adopted in this study were designed to assess impairments consistent with the known functional architecture of the rat brain.

**Figure 1:**
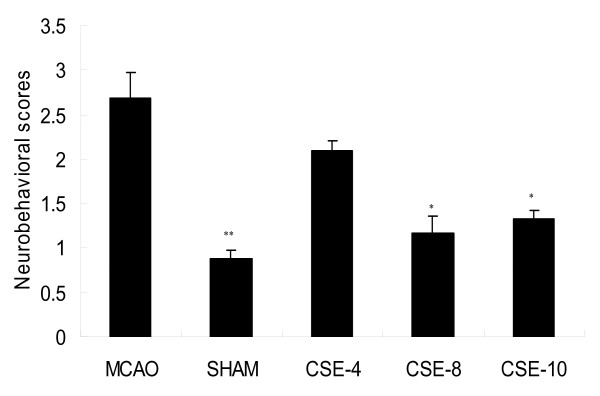
**Effect of CSE on the Development of Behavioral Abnormalities after middle cerebral artery occlusion **. Values are shown as means ± SEM. *p < 0.05 vs. MCAO group, **p < 0.01 vs. MCAO group. (F_SHAM _= 17.642, df_SHAM _= 11, p_SHAM _= 0.001, F _CSE-4 _= 2.252, df _CSE-4 _= 11, p _CSE-4 _= 0.162, F _CSE-8 _= 16.396, df _CSE-8 _= 11, p _CSE-8 _= 0.01, F _CSE-10 _= 22.738, df_SHAM _= 11, p_SHAM _= 0.02)

Twenty-four hours after MCAO in rats, neurological deficit scores were significantly reduced in CSE-8 -treated rats and CSE-10 -treated rats. The neurobehavior for the SHAM group was 0.9 (0.6--1.1), the MCAO group was 3.7 (2.6--5.3), the CSE-4 group was 2.2 (1.8--4.1), the CSE-8 group was 1.2 (0.8--4.1) and the CSE-10 group was 1.5 (1.0--4.1). It is clear that the behavioral abnormality was significantly developed in the MCAO group as compared with the sham (Figure 1). In contrast, the CSE-8 group and CSE-10 significantly suppressed the development of behavioral abnormality as compared with the MCAO group (Figure 1).

The grip strength in the SHAM group was found to be 0.963 ± 0.006 kg units. A significant decrease in the grip strength was observed in the MCAO group, as compared to the sham rats (*P *< 0.01). CSE-8 and CSE-10 treated rats showed a significant increase in grip strength, as compared to the MCAO group (*P *< 0.01) (Table 1).

**Table 1:** Effect of CSE on basal grip strength

Different groups	Grip strength (Kg Units)	F-values	df-values	p-values
MCAO group	0.598 ± 0.004 ^b^			
SHAM group	0.963 ± 0.006 ^a^	0.158	11	0.001
CSE-4 group	0.663 ± 0.022^b^	0.158	11	0.445
CSE-8 group	0.978 ± 0.005^a^	0.146	11	0.001
CSE-10 group	0.890 ± 0.013^a^	2.964	11	0.005

Values are shown as means ± SEM. The different letters in the same column indicate a statistical difference (*p *< 0.01 vs. MCAO group).

Increasing evidence has indicated that ischemia/reperfusion occurs due to oxidative stress that may potentiate ischemic injury [32]. Lactate dehydrogenase was measured to evaluate the role of antioxidative stress in the protection of CSE. The serum LDH levels in SHAM group were found to be 86.322 ± 2.663 IU/L. A significant increase in the activity of LDH in serum was observed in MCAO group, as compared to the SHAM group; whereas, CSE-8 and CSE-10 treatment significantly resulted in decreased serum LDH levels when compared with MCAO group rats (Table 2).

**Table 2:** Effect of CSE on serum LDH levels

Different groups	LDH (IU/L)	F-values	df-values	p-values
MCAO group	170.201 ± 3.741			
SHAM group	86.322 ± 2.663***	6.556	5.183	0.000
CSE-4 group	156.601 ± 2.221	7.199	11	0.118
CSE-8 group	106.632 ± 3.111**	7.497	5.063	0.001
CSE-10 group	131.232 ± 2.101**	0.426	11	0.005

Values are shown as means ± SEM. **p < 0.01 vs. MCAO group, ***p < 0.001 vs. MCAO group

Reduced glutathione (GSH) is one of the primary endogenous antioxidant defense systems in the brain, which removes hydrogen peroxide and lipid peroxides. Decline in GSH levels could either increase or reflect oxidative status [33,34]. Concentrations of GSH were lower in MCAO group than those in SHAM group (Table 3 and Table 4). CSE-8 produced the increase in the level of GSH significantly. The same results did occur in the CSE-10 group.

**Table 3:** Effect of CSE on Hippocampus GSH

Different groups	Hippocampus GSH (nmol GSH/mg protein)	F-values	df-values	p-values
MCAO group	0.763 ± 0.025			
SHAM group	2.298 ± 0.013**	7.992	6.285	0.009
CSE-4 group	1.015 ± 0.015	1.863	11	0.379
CSE-8 group	2.465 ± 0.055**	6.537	6.406	0.008
CSE-10 group	1.233 ± 0.022*	2.964	11	0.025

Values are shown as means ± SEM. *p < 0.05 vs. MCAO group, **p < 0.01 vs. MCAO group

**Table 4:** Effect of CSE on Cerebral cortex GSH

Different groups	Cerebral cortex GSH (nmol GSH/mg protein)	F-values	df-values	p-values
MCAO group	1.012 ± 0.010			
SHAM group	1.803 ± 0.026*	4.193	11	0.017
CSE-4 group	1.095 ± 0.021	4.043	11	0.336
CSE-8 group	1.495 ± 0.053*	3.306	11	0.026
CSE-10 group	1.295 ± 0.011*	3.418	11	0.427

Values are shown as means ± SEM. *p < 0.05 vs. MCAO group

It can be attributed to several factors such as cleavage GSH to cysteine, decrease in the synthesis of GSH and the formation of mixed disulfides, causing their cellular stores to be depleted [35].

The large numbers of polyunsaturated fatty acids make cell membranes particularly vulnerable to lipid peroxidation. The oxidation of polyunsaturated fatty acids alters the structure of the membrane with resultant changes in fluidity and permeability. Lipid peroxidation can also inhibit the function of membrane bound receptors and enzymes [36]. The level of LPO content adds to the proof of the increased peroxidative damage during cerebral ischemia. A significant increase in the content of LPO was observed in the MCAO group when compared with the SHAM group. In the CSE-8 and CSE-10 group, a significant decrease was seen in the level of LPO when compared with the MCAO group (Table 5).

**Table 5:** Effect of CSE on LPO level

Different groups	nmol LPO/g protein	F-values	df-values	p-values
MCAO group	20.21 ± 1.41			
SHAM group	13.23 ± 0.66**	5.254	6.996	0.003
CSE-4 group	20.01 ± 0.21	19.460	5.010	0.846
CSE-8 group	15.32 ± 0.11*	1.304	10	0.043
CSE-10 group	15.22 ± 0.21*	1.305	10	0.042

Values are shown as means ± SEM. *p < 0.05 vs. MCAO group, **p < 0.01 vs. MCAO group

It has been proposed that antioxidant changes reflect an altered redox balance in several pathological states. The antioxidants would be consumed in the reaction with free radicals. Therefore, the measurement of endogenous antioxidants enzymes i.e. GPx, GR, CAT and GST as well as Na^+^K^+^ATPase has been performed to estimate the amount of oxidative stress. Activities of various antioxidant enzymes and Na^+^K^+^ATPase of different groups have been listed in Table 6, Table 7, Table 8, Table 9 and Table 10. The activity of endogenous antioxidant enzymes was decreased significantly in the MCAO group, as compared to the sham group, whereas in the CSE-8 group, CSE-treatment showed a significant restoration in the level of various enzymes as compared with MCAO group. The same results did occur in the CSE-10 group.

**Table 6:** Effect of CSE on the activity of GPx

Different groups	GPx	F-values	df-values	p-values
MCAO	8.01 ± 0.42			

SHAM	15.98 ± 1.23**	0.714	11	0.001
CSE-4	9.10 ± 1.02	0.911	11	0.306
CSE-8	12.16 ± 1.32**	3.557	11	0.006
CSE-10	10.10 ± 1.11*	0.052	11	0.04

Values are shown as means ± SEM. *p < 0.05 vs. MCAO group, **p < 0.01 vs. MCAO group

**Table 7:** Effect of CSE on the activity of GR

Different groups	GR	F-values	df-values	p-values
MCAO	20.88 ± 2.11			

SHAM	35.55 ± 2.51**	0.953	11	0.006
CSE-4	21.01 ± 2.02	0.03	11	0.954
CSE-8	29.01 ± 2.22*	4.394	11	0.028
CSE-10	25.00 ± 2.31*	4.929	11	0.048

Values are shown as means ± SEM. *p < 0.05 vs. MCAO group, **p < 0.01 vs. MCAO group

**Table 8:** Effect of CSE on the activity of GST

Different groups	GST	F-values	df-values	p-values
MCAO	10.07 ± 1.11			

SHAM	15.00 ± 1.22**	16.088	13	0.002
CSE-4	10.60 ± 0.97	9.946	10	0.680
CSE-8	13.60 ± 0.98**	19.214	11	0.008
CSE-10	12.60 ± 0.88*	20.293	12	0.029

Values are shown as means ± SEM. *p < 0.05 vs. MCAO group, **p < 0.01 vs. MCAO group

**Table 9:** Effect of CSE on the activity of CAT

Different groups	CAT	F-values	df-values	p-values
MCAO	4.22 ± 0.13			

SHAM	6.11 ± 0.33*	7.196	12	0.010
CSE-4	4.78 ± 0.24	3.917	10	0.076
CSE-8	5.78 ± 0.23*	6.977	11	0.02
CSE-10	5.70 ± 0.30*	6.858	11	0.024

Values are shown as means ± SEM. *p < 0.05 vs. MCAO group

**Table 10:** Effect of CSE on the activity of Na^+^K^+^ATPase

Different groups	Na^+^K^+^ATPase	F-values	df-values	p-values
MCAO	2.00 ± 0.13			
SHAM	4.52 ± 0.32**	16.967	11	0.002
CSE-4	2.11 ± 0.10	0.175	11	0.684
CSE-8	3.11 ± 0.11*	17.788	11	0.001
CSE-10	4.13 ± 0.22*	16.302	11	0.002

Values are shown as means ± SEM. *p < 0.05 vs. MCAO group, **p < 0.01 vs. MCAO group

A great deal of effort has been directed toward searching for a new drug that can be used for protection of cerebral ischemia-reperfusion injury. From the point of view of "self-medication" or "preventive medicine," *Cordyceps sinensis *was used in the prevention of cerebral ischemia in this paper. Although *Cordyceps sinensis *is extensively used in Chinese medicine, it lacks scientific grounds for its efficacy and to the best of our knowledge this is the first study to report its possible protective mechanisms against cerebral ischemic damage.

Here we showed that the *Cordyceps sinensis *extract significantly improved the outcome in rats after cerebral ischemia and reperfusion in terms of neurobehavioral function. At the same time, supplementation of *Cordyceps sinensis *extract significantly boosted the defense mechanism against cerebral ischemia by increasing antioxidants activity related to lesion pathogenesis. Restoration of the antioxidant homeostasis in the brain after reperfusion may have helped the brain recover from ischemic injury.

## Conclusions

These experimental results suggest that complement *Cordyceps sinensis *extract is protective after cerebral ischemia in specific way. The administration of CSE significantly reduced focal cerebral ischemic/reperfusion injury. The defense mechanism against cerebral ischemia was by increasing antioxidants activity related to lesion pathogenesis.

## Competing interests

The authors declare that they have no competing interests.

## Authors' contributions

ZL conceived of the study, and participated in its design and coordination. PL participated in the operation of the study. DZ participated in the statistical analysis. HT carried out the preparation of CSE. JG drafted the manuscript. All authors read and approved the final manuscript.
